# Investigating the complexity of respiratory patterns during the laryngeal chemoreflex

**DOI:** 10.1186/1743-0003-5-17

**Published:** 2008-06-20

**Authors:** Andrei Dragomir, Yasemin Akay, Aidan K Curran, Metin Akay

**Affiliations:** 1 Harrington Department of Bioengineering, Ira A. Fulton School of Engineering Arizona State University, Tempe, AZ 85287, USA; 2 Department of Physiology, Dartmouth Medical School, NH 03756, USA

## Abstract

**Background:**

The laryngeal chemoreflex exists in infants as a primary sensory mechanism for defending the airway from the aspiration of liquids. Previous studies have hypothesized that prolonged apnea associated with this reflex may be life threatening and might be a cause of sudden infant death syndrome.

**Methods:**

In this study we quantified the output of the respiratory neural network, the diaphragm EMG signal, during the laryngeal chemoreflex and eupnea in early postnatal (3–10 days) piglets. We tested the hypothesis that diaphragm EMG activity corresponding to reflex-related events involved in clearance (restorative) mechanisms such as cough and swallow exhibit lower complexity, suggesting that a synchronized homogeneous group of neurons in the central respiratory network are active during these events. Nonlinear dynamic analysis was performed using the approximate entropy to asses the complexity of respiratory patterns.

**Results:**

Diaphragm EMG, genioglossal activity EMG, as well as other physiological signals (tracheal pressure, blood pressure and respiratory volume) were recorded from 5 unanesthetized chronically instrumented intact piglets. Approximate entropy values of the EMG during cough and swallow were found significantly (*p *< 0.05 and *p *< 0.01 respectively) lower than those of eupneic EMG.

**Conclusion:**

Reduced complexity values of the respiratory neural network output corresponding to coughs and swallows suggest synchronous neural activity of a homogeneous group of neurons. The higher complexity values exhibited by eupneic respiratory activity are the result of a more random behaviour, which is the outcome of the integrated action of several groups of neurons involved in the respiratory neural network.

## Background

The laryngeal chemoreflex (LCR) has been investigated in many epidemiological and physiological studies as a putative exogenous stressor that may contribute to the pathogenesis of sudden infant death syndrome (SIDS) [1-3]. The triple-risk model proposed for SIDS states that death occurs at the confluence of three factors – a inherently vulnerable infant, exposed to an exogenous stressor during a critical period of postnatal development [4]. The LCR is elicited when liquid reaches the laryngeal mucosal receptors. Commonly, the LCR response consists of a series of events that may be categorized as *conservative *(in terms that they try to preserve the limited oxygen reserves without removing the reflex causing stimulus) such as apnea, bradycardia and redistribution of blood flow or *restorative *(events that try to clear the stimulus and restore the normal functioning of the airway): swallowing and coughing [1]. Previous studies have suggested that while swallowing and apnea are predominant in the postnatal period, cough emerges as a stronger response as the animals develop into adulthood [5].

The manifestations of LCR consist of swallowing and coughing, which occur frequently, apnea (usually associated with bradycardia), startle, laryngeal constriction and arousal from sleep. Swallowing and coughing are the primary manifestations, while the others may or may not appear depending on the type and strength of the stimulus. Apneas usually follow a period of swallowing and coughing, while coughing is usually associated with prior arousal. Swallowing and coughing remove fluids from the pharyngeal airway, while apnea combined with the laryngeal constriction prevent aspiration. Generally the conservative and restorative aspects of the reflex are mutually exclusive [1]. Prolonged apneas pose paradoxically a great danger: even if together with the resultant hypoxia and bradycardia they are part of a preventive mechanism, they might become lethal if the system is not restored in a timely manner [5]. Previous studies indicated apnea duration to be strongly influenced by the stimulus type (water being much more effective than saline solutions) but even more by a central neural mechanism that perpetuates respiratory depression, altered central neural processing of receptor input being a highly relevant factor [6]. The whole LCR duration was found to be prolonged by vulnerabilities of the neurons in the rostral ventral medulla (RVM) and to enhance the disruption of stable respiratory patterns within this context, thus strengthening its relevance in SIDS [1].

In recent studies we have investigated the complexity of respiratory patterns during eupnea and hypoxia using nonlinear dynamic analysis and time-frequency analysis of the phrenic neurogram during early maturation [7,8]. Our results suggested that during severe hypoxia (gasping) the complexity of the respiratory neural networks is reduced and this might be due to the silencing of neurons responsible for activities in the early phase of the phrenic neurogram.

In the current study, we aim at gaining insight into the output of the respiratory network in piglets during the LCR and assess the changes in respiratory patterns complexity during cough, swallow and early recovery after apnea, when compared to eupnea. We aim at proving that during the LCR the activity of the respiratory neural networks is taken over by a homogeneous group of neurons; hence we should observe reduced complexity in the respiratory patterns during the key restorative events. Obviously, vulnerability within some of these neurons might be fatal.

Quantitative changes in the complexity of biomedical signals have been traditionally assessed using nonlinear dynamics analysis methods [9-11]. Generally, physiological signals are complex and thought to originate from complex nonlinear systems [12-14]. Since respiratory motor output depends on the integrated properties of the central respiratory neural network, and such a system has complex dynamic behaviour, the respiratory patterns present irregular (complex) features that reflect the dynamics of the underlying neural network. Therefore, nonlinear dynamics methods have been preferred to spectral analysis and time domain or time-frequency analysis methods in the cases when information about the system generating the output is needed [11].

The approximate entropy (ApEn), which is a method commonly used to asses irregularity (complexity) of biological signals [7,15], was chosen for our analysis. Since many biological signals have short data length (100–5000 points) and traditional nonlinear dynamics analysis methods are largely dependent on the length of the data sequence [15,16], the approximate entropy method has been proposed as an ideal tool for these cases [17]. ApEn is computationally efficient and produces accurate estimates in the case of short data segments.

## Methods

### Experiments

Experiments were performed on 5 unanesthetized chronically instrumented intact piglets ranging in age from 3 to 10 days. All experimental protocols and surgeries were approved by The Institutional Animal Care and Use Committee of Dartmouth College. Animals were anesthetized using isoflurane in O_2_. Two-wire electro-myographic (EMG) electrodes were sewn into the diaphragm through a subcostal incision in the right upper quadrant of the abdomen to monitor the diaphragm EMG (EMGdia) activity. Another set of wires was inserted into the genioglossus through a submental incision to monitor genioglossal EMG (EMGgg) activity. A 2.7 mm-diameter catheter was placed in the trachea just below the cricoid cartilage to record endotracheal pressure and exteriorized between the shoulder blades on the animals back. The wires were tunneled subcutaneously and exited the skin at the top of the skull. Respiration was measured by using a barometric plethysmograph modified to allow continuous gas flow [1]. A dual-lumen umbilical catheter was inserted into the femoral artery, with one lumen connected to a transducer to measure arterial BP, while the second lumen was used to withdraw blood-gas samples. To stimulate the LCR, a pharyngeal catheter was placed through the nose at the time of experiments. EEG electrodes were screwed into the skull over the left frontal, right occipital and right parietal regions, while EOG electrodes were placed lateral to and just above each eye. A pair of EMG wires was placed in the neck muscles posteriorly. EEG, EOG and neck EMG were used to determine animals arousals and sleep states. These wires were also exteriorized at the top of the skull and, along with diaphragm anf GG EMG wires, were attached to brass connectors and placed in a plastic connector. The connector was sealed and attached to the skull with acrylic adhesive. The connector could be attached to recording leads to acquire data from conscious animals.

The animals were studied ~24 h after the surgery. The EMGdia and EMGgg were amplified and band- pass filtered from 10–300 Hz. Respiration, endotracheal pressure, blood pressure and animal temperatures were recorded continuously. All signals were sampled at 1000 Hz and recorded using a data acquisition system (PowerLab, ADInstruments).

### LCR characterization

**Figure 1:**
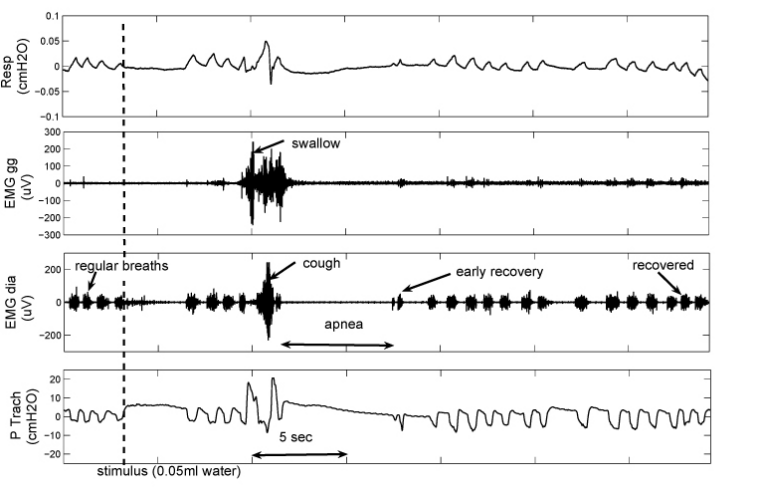
**Typical tracings during the LCR in a 10 days old piglet**. Example of the events undergoing during the LCR. After the stimulus, swallowing is visible on the EMGgg tracing and coughing is visible on the EMGdia. Apnea results in the cessation of respiratory activity and this is visible on all channel.

Figure 1 displays some typical signal tracings during the LCR. Coughing was detected by a massive increase in EMGdia activity that preceded forceful expiratory activity, easily identifiable by an increase on the tracheal pressure tracing [1]. Swallow was associated with a negative deflection on the tracheal pressure tracing and a burst visible on the EMGgg. Apneas were defined as periods of silence on the EMGdia and EMGgg (reflecting no breathing activity) that lasted longer than the last 2 normal breaths before the moment of stimulus application. Early recovery breaths are considered the first bursts visible on the EMGdia following the apnea and they are the outcome of the systems' efforts to restore normal activity. The end of the LCR was considered when 5 regular (eupneic) consecutive breath bursts were observed. Generally, apnea occurs after coughing and swallowing activities, which appear at the onset of LCR. These manifestations of the reflex are mutually exclusive. There was no coughing observed without prior arousal. Arousal was identified by characteristic small amplitude, high frequency EEG tracings, as well as large amplitude bursts on the EOG and increased activity on the neck EMG.

### Approximate entropy

The approximate entropy is a statistical measure that smooths transient interference and can suppress the influence of noise by properly setting of the algorithms parameters. It can be employed in the analysis of both stochastic and deterministic signals [17,18]. This is crucial in the case of biological signals, which are outputs of complex biological networks and may be deterministic or stochastic, or both. ApEn provides a model-independent measure of the irregularity of the signals. The algorithm summarizes a time series into a non-negative number, with higher values representing more irregular systems [17,18].

The approximate entropy estimates are calculated using segments *X*(*i*) through *X*(*N *- *m *+ 1) defined by *X*(*i*) = [*x*(*i*), ..., *x*(*i *+ *m *- 1)]. The difference between *X*(*i*) and *X*(*j*), *d *[*X*(*i*), *X*(*j*)] as the maximum absolute difference between their related scalar elements can be estimated as:

(1) *d *[*X*(*i*), *X*(*j*)] = max_*k *= 0,*m*-1 _[|*x*(*i *+ *k*) - *x*(*j *+ *k*)] ≤ *r*

assuming that all the differences between the corresponding elements will be less than the threshold *r*.

For any given *X*(*i*), the ratio of the difference between *X*(*i*) and *X*(*j*) smaller than the threshold *r *to the total number of vectors (*N *- *m *+ 1) is obtained as:

(2) $$\begin{array}{cc} C_{r}^{m}(i)=N_{r}^{m}(i)/(N-m+1) & \begin{array}{cc} for & i=1,...,N-m+1 \end{array} \end{array}$$

The approximate entropy, ApEn(*m*,*r*), can be estimated as a function of the parameters *m *and *r *as follows:

(3) $$ApEn(m,r)=\underset{N\to \infty }{\lim}\left[\Phi^{m}(r)-\Phi^{m+1}(r)\right]$$

where

(4) $$\Phi^{m}(r)=\sum_{i=1}^{N-m+1}\ln C_{r}^{m}(i)/(N-m+1)$$

In practice, the approximate entropy values can be estimated for a signal with *N *samples as:

(5) ApEn(*m*, *r*, *N*) = [*Φ*^*m *^(*r*) - *Φ*^*m*+1 ^(*r*)]

The parameter *m *is the embedding dimension of the analyzed signals and the parameter *r *is the threshold to suppress the noise in the signal. Throughout this study we have chosen *m *= 2 as described in previous works [11,17,18]. The parameter *r *can be chosen as 0.1SD(*x*(*i*)), where SD(*x*(*i*)) represents the standard deviation of the original signal *x*(*i*).

## Results

Our objective in this study was the investigation of changes in the complexity of the central respiratory network of the piglets during the LCR. EMGdia, EMGgg as well as other physiological signals needed to completely characterize the manifestations of the reflex were recorded. 5 piglets, aged 3–10 days, were used for the experiments. The LCR was elicited by injecting 0.05 ml water into the larynx via a nasal catheter. The recorded signals were detrended by removing their mean before analysis using ApEn was performed.

The respiratory volume, EMGgg, EMGdia and tracheal pressure recordings corresponding to a reflex elicited in a 10 days old piglet are shown in Figure 1. Totally, the reflex lasts ~30 sec; the water stimulus first triggers the swallow, which is immediately followed by a cough and afterwards apnea. Apnea duration is ~6 sec, with the system subsequently attempting to recover. There are several early recovery breaths which show a characteristic pattern. They have shorter duration than regular breaths and their early phase (first half) activity seems decreased, resembling patterns in gasping following hypoxia [7]. Regular respiratory activity is restored after ~30 sec, counted when 5 consecutive regular breaths appear [1]. In the presented case swallowing precedes the cough but during the experiments we observed swallows also succeeding the cough as well as after apnea.

To investigate how the complexity of respiratory patterns change during the LCR, we split the respiratory patterns into 5 characteristic groups: regular (eupneic) breaths (breaths occurring before the stimulus was given), swallows, coughs, early recovery breaths (first breath burst visible on the EMGdia after apnea) and recovered breaths (at least 5 consecutive breaths similar to the regular breaths before stimulus but occurring not earlier than 25 sec after the stimulus). The latter condition was imposed based on observations from previous studies which determined an average duration of 20–25 sec for the water-elicited LCR in early postnatal piglets [1].

**Figure 2:**
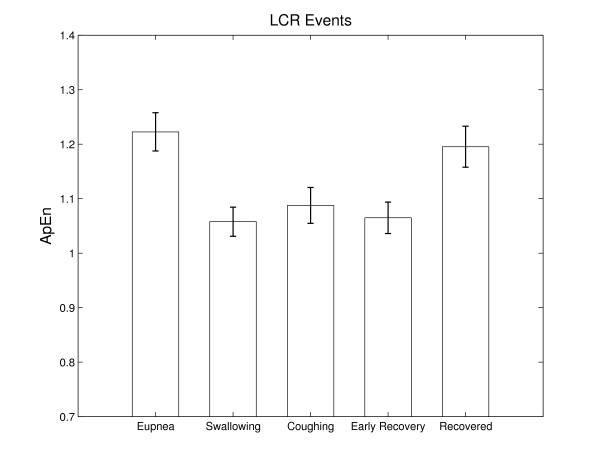
**Approximate entropy values during LCR**: Approximate entropy values ± standard error for the 5 characteristic groups of respiratory patterns during the LCR: regular (eupneic) breaths, swallowing, coughing, early recovery breaths following apnea and fully recovered breaths. Results represent averages of entropy values of 3 measurements for each of the 5 animals under study.

Figure 2 displays the average approximate entropy (complexity) values measured for the 5 piglets under study. The values represent means ± standard error of 3 separate measurements for each subject, corresponding to 3 elicited reflexes. It is easily observable that the complexity values are highest in the case of regular (eupneic) breaths; the recovery breaths have values similar to the regular ones, indicating that the system restored its normal functioning after the reflex. Swallow and cough bursts, despite their longer time duration exhibit very low complexity values, indicating that respiratory networks' output during these events are the result of the activity of a homogenous group of neurons. Early recovery breaths show relatively low values too, which might be an indication that the preceding apnea silences some groups of neurons within the central respiratory network. The generally observed trend throughout our experiments was that the first breath following apnea had the lowest entropy value, while subsequent breaths exhibited continuously increasing values. Generally, after ~30 sec the entropy of the breaths return to values comparable to those before the stimulus application.

We used the analysis of variance (ANOVA) to compare the significance of the differences in the means of the resulting approximate entropy values. Thus, swallows had significantly lower values than regular breaths (*p *< 0.01), and also than recovered breaths (*p *< 0.05). Coughs had significantly lower values (*p *< 0.05) than regular and recovered breaths. Early recovery breaths were significantly different when compared to the regular breaths (*p *< 0.01) and fully recovered breaths (*p *< 0.05), but fully recovered breaths complexity values were not significantly different when compared to the regular breaths (*p *> 0.1).

**Figure 3:**
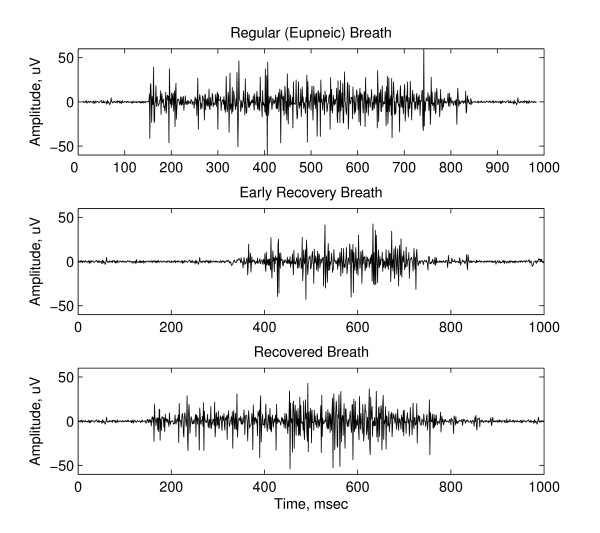
**Typical EMGdia tracings for eupneic, early recovery and recovered breaths**. EMGdia tracings corresponding to regular breathing activity (top plot), early recovery breath, following apnea (middle plot) and a fully recovered breath (bottom plot) of a 10 days old piglet.

To further characterize the changes undergone by the respiratory patterns during the LCR, we have also studied the breathing temporal patterns. Figure 3 presents comparatively the EMGdia tracings of a 10 days old piglet. The top plot corresponds to a regular eupneic breath occurring before the application of the LCR stimulus (0.05 ml water solution). The middle plot shows an early recovery breath, occurring immediately after an apnea period. The bottom plot presents a breath occurring ~30 sec after the stimulus application. All three plots correspond to the same induced LCR.

The shorter duration of the burst and the signal shape in the middle plot, resembles those of hypoxic bursts (gasping) studied in our previous works [7,8]. The resemblance to gasping extends to the fact that early recovery breaths following apnea are characterized by brief, intense inspiratory efforts of the diaphragm and other respiratory muscles. Previous studies agreed that gasping is the result of a unique medullary pattern generator which does not contribute to eupneic breathing [19]. We hypothesize (and intend to test this hypothesis in future studies) that the mechanism responsible for the respiratory activity during early recovery might be similar to the one involved in gasping, where all inspiratory neurons fire simultaneously at the beginning of the inspiratory period [19]. On the other hand, the pattern exhibited in the bottom plot highly resembles the one on the top plot, indicating systems' full recovery after the critical respiratory disruption associated to the LCR.

Furthermore, Table 1 displays average durations (means ± standard errors) of LCR related events and of eupneic breaths for the 5 piglets under study. The events are the same ones that were considered for the approximate entropy estimations presented above. The results reinforce the previous observations, suggesting that the early recovery breaths occurring after apnea exhibit shorter duration possibly due to an apnea-influenced mechanism that silences part of the neural activity. We interpret these respiratory efforts as a last resort attempt of the system to restore normal activity. As expected, breaths occurring after recovery from LCR have similar durations with regular eupneic breaths. Coughing and swallowing have significantly longer durations. Another interesting observation is that older animals (8 and 10 days) exhibit longer duration of respiratory activities, when compared to younger ones (3 days), results that agree with previous studies that investigated changes in the respiratory system in the context of early maturation [5,9]. This is due to the fact that respiratory premotor and motor neurons undergo rapid changes in biochemical and bioelectric properties during the first month of postnatal life. Thus, there is an increase in the complexity of the dendritic tree of respiratory neurons as it changes from a bipolar to a multipolar morphology [20,21].

**Table 1:** Average durations (in msec) of respiratory activity during the laryngeal chemoreflex 5 piglets, 3–10 days old, for each piglet the reflex was elicited 3 times.

LCR EVENTS					
Piglet	Regular breath	Swallowing	Coughing	Early recovery	Recovered breath
1 (3 days)	490.66 ± 20.34	728.33 ± 40.03	891.66 ± 27.79	358.00 ± 16.06	474.66 ± 14.37
2 (3 days)	472.33 ± 22.16	775.00 ± 23.43	862.33 ± 33.67	381.33 ± 19.81	462.33 ± 17.23
3 (3 days)	563.00 ± 16.18	801.66 ± 18.40	899.00 ± 21.62	392.66 ± 23.16	570.33 ± 20.04
4 (8 days)	641.66 ± 31.26	910.33 ± 32.84	991.33 ± 41.28	401.33 ± 20.55	621.00 ± 28.43
5 (10 days)	766.00 ± 24.78	926.33 ± 34.15	1004.66 ± 38.44	441.66 ± 18.22	767.33 ± 23.81

Mean	586.73 ± 29.34	828.33 ± 23.71	929.79 ± 34.82	394.99 ± 21.63	576.13 ± 26.25

## Discussion and conclusion

Coughing and swallowing are part of a defense mechanism that develops in fetus and continues in postnatal life aiming to protect the airway from fluid ingestion. Failure of these mechanisms might result in life threatening conditions. Apnea plays also an important role in preserving the limited oxygen resources, without, however, removing the offending stimulus. Paradoxically, prolonged apnea resulting from vulnerabilities within groups of neurons in the central respiratory network might be fatal [5]. Our results support this supposition, the early recovery breaths after apnea presenting significantly reduced complexity values and shorter duration than regular breaths, suggesting that apnea silences part of the neural activity via a mechanism that might be similar with that involved in gasping [7,8].

Reduced complexity values of the respiratory neural network output corresponding to coughs and swallows suggest synchronous neural activity of a homogeneous group of neurons that might be taking over respiratory activity under emergency conditions. The higher complexity values exhibited by eupneic respiratory activity are the result of a more random behavior, which is the outcome of the integrated action of several groups of neurons involved in the respiratory neural network.

The whole succession of events aiming at protecting the laryngeal airway is commonly known as the laryngeal chemoreflex (LCR). It involves coughing, swallowing, apnea, laryngeal constriction, startle and bradycardia. Our findings suggest that respiratory patterns show significantly reduced complexity throughout the duration of LCR. This poses the organism under great threat when combined with an underlying neural vulnerability and in conjunction with failed cardiorespiratory and arousal responses to physiological stimuli often encountered during early maturation. This supports the results of previous studies indicating LCR as part of the risks associated to sudden infant death syndrome (SIDS) [1,5].

## Competing interests

The authors declare that they have no competing interests.

## Authors' contributions

AD performed the data processing and analysis and drafted the manuscript, YA participated in the study design and results interpretation, AKC performed the experiments, MA guided the data procesisng and analysis, interpreted the results and contributed to writing the manuscript.
